# ﻿Multi-locus molecular phylogenetic analysis reveals two new species of *Amphichorda* (Bionectriaceae, Hypocreales)

**DOI:** 10.3897/mycokeys.106.117205

**Published:** 2024-07-03

**Authors:** Zhi-Qin Wang, Jing Zhao, Quan-Ying Dong, Yao Wang, Ying-Ling Lu, Run Luo, Hong Yu

**Affiliations:** 1 Yunnan Herbal Laboratory, College of Ecology and Environmental Sciences, Yunnan University, Kunming, Yunnan, China Yunnan University Kunming China; 2 The International Joint Research Center for Sustainable Utilization of Cordyceps Bioresources in China and Southeast Asia, Yunnan University, Kunming, Yunnan, China Yunnan University Kunming China

**Keywords:** Coprophilous fungi, diversity, morphology, new taxa, taxonomy

## Abstract

*Amphichorda* has been previously accepted as a member of the Cordycipitaceae and currently it is considered a member of the Bionectriaceae. The substrates of *Amphichorda* were complex and varied, being mainly animal faeces. This study reports two new species of *Amphichorda* from Yunnan Province in south-western China. Based on the five-gene (nr*SSU*, nr*LSU*, *tef‐1α*, *rpb1* and *rpb2*) sequence and ITS data phylogenetic analysis, two new species, namely *A.excrementa* and *A.kunmingensis*, are proposed and a detailed description of the new species is provided. *Amphichordaexcrementa* and *A.kunmingensis* were isolated from animal faeces in the park. The morphological characteristics of two novel species and seven known species in *Amphichorda* are also compared.

## ﻿Introduction

*Amphichorda* Fr. was established to accommodate the type species *A.felina* (DC.) Fr., which was isolated from cat dung and previously classified in the genus *Clavaria* (Lamarck 1815; Fries 1825). At the present, seven species of the *Amphichorda* are now validly published (Zhang et al. 2017, 2021; Guerra-Mateo et al. 2023; Liu et al. 2023; Leão et al. 2024). The traditional phylogenetic placement of the genus *Amphichorda* was considered in the family Cordycipitaceae (Hypocreales). The Cordycipitaceae is the most complex group in the order Hypocreales because of its varied morphological characteristics and wide-ranging hosts and some genera present numerous taxonomical problems (Wang et al. 2020; Guerra-Mateo et al. 2023). In the studies of Zhang et al. (2017, 2021) and Liu et al. (2023) which report new species of the genus *Amphichorda*, the phylogenetic position of *Amphichorda* belongs to the Cordycipitaceae. However, Guerra-Mateo et al. (2023) conducted the phylogenetic analysis based on the nuclear ribosomal internal transcribed spacer region (ITS) and the nuclear ribosomal large subunit (nr*LSU*), considered *Amphichorda* to belong to the family Bionectriaceae and determined *Amphichorda* has close phylogenetic relationships with the genera *Hapsidospora* and *Nigrosabulum*. Leão et al. (2024) also proving the genus *Amphichorda* belongs to the family Bionectriaceae.

The taxonomic status of the type species has been controversial since the original description of the type species of the *Amphichorda*. *Amphichordafelina* was classified as *Beauveria* in 1980 (Carmichael et al. 1980). However, early phylogenetic analyses showed that *Beauveriafelina* was distant from other *Beauveria* species and that it was morphologically distinguished from other *Beauveria* species by the absence of elongate conidiogenous cells with apical denticulate rachis (Rehner et al. 2011; Zhang et al. 2017; Liu et al. 2023). The type strain of *A.felina* (= *B.felina*) seems to be unknown (Guerra-Mateo et al. 2023). *Isariacretacea* J.F.H. Beyma type strain CBS 250.34 was considered to be the type strain of *A.felina* since *I.cretacea* was synonymised with *A.felina* (De Hoog 1972; Zhang et al. 2021; Guerra-Mateo et al. 2023). However, the criteria required for fungal epitypification were the substrate and geographic similarity (Guerra-Mateo et al. 2023). The substrate and geography were different between *A.felina* and the strain CBS 250.34, so this strain has not been designated as the epitype of *A.felina* (Lendemer 2020). Guerra-Mateo et al. (2023) proposed that the strain CBS 250.34 can be accepted as a reference to stabilise the nomenclature of *A.felina*, but should be avoided to indicate it as a type strain of *A.felina* (Zhang et al. 2017, 2021; Wang et al. 2020; Liu et al. 2023).

During the surveys of entomopathogenic fungifrom two regions in Yunnan Province, China, the animal faeces were collected and three strains were isolated from the specimens. Based on morphological evidence together with the five-gene (nr*SSU*, nr*LSU*, *tef‐1α*, *rpb1* and *rpb2*) sequence and ITS data analyses of some genera in Bionectriaceae, it was shown that the three strains belong to the genus *Amphichorda*. On the basis of its morphological characteristics and multi-locus molecular phylogenetic analyses, two new species were described. Furthermore, the morphological characteristics of two novel species and seven known species in *Amphichorda* were compared.

## ﻿Materials and methods

### ﻿Fungal collection and isolation

The specimens were collected in Kunming City, Yunnan Province, China in July 2019. In the field, it was placed in sterilised plastic pipes and brought to the laboratory for isolation. In order to obtain axenic cultures, part of the surface tissue of the specimen was cut off with a sterilised dissecting knife and then placed into a flask containing 10 ml of sterilised water and glass beads. Then the suspension was shaken for 10 min and diluted 50 times. Finally, the diluted suspension was applied on Petri dishes with potato dextrose agar (PDA: fresh potato 200 g/l, dextrose 20 g/l and agar 18 g/l) containing 0.1 g/l streptomycin and 0.05 g/l tetracycline. Then the Petri dish was placed in a room at 15 °C to allow it to grow, during which time the growing fungiwere transferred one by one to new Petri dishes. After isolation into pure cultures, they were transplanted to a PDA slant and stored at 4 °C. The specimens were deposited in the Yunnan Herbal Herbarium (YHH) of Yunnan University, China. The strain was deposited at the Yunnan Fungal Culture Collection (YFCC) of Yunnan University, China. The culture of the *Amphichordafelina* (CBS 250.34) was obtained from the culture collection (CBS) of the Westerdijk Fungal Biodiversity Institute (WI) in Utrecht, the Netherlands. The obtained strain CBS 250.34 was inoculated into PDA medium and re-cultured.

### ﻿Morphological observations

Colonies were incubated on PDA for three weeks in an incubator at 25 °C. The photograph was taken morphologically using a Canon 750 D camera (Canon Inc., Tokyo, Japan). The anamorphs (Conidiophores, Phialides and Conidia) in culture were observed using a light microscope (Olympus BX53). The growth rate of colonies was calculated using the method of Liu and Hodge (2005) and it was categorised as: fast-growing (30–35 mm in diameter), moderately growing (20–30 mm in diameter) and slow-growing (< 20 mm in diameter).

### ﻿DNA extraction, PCR and sequencing

The genomic DNA was extracted from axenic living cultures using the Genomic DNA Purification Kit (Qiagen GmbH, Hilden, Germany) according to the manufacturer’s instructions. The five-gene (nr*SSU*, nr*LSU*, *tef‐1α*, *rpb1* and *rpb2*) and ITS were sequenced and the following primer pairs were used for PCR amplification. The nuclear ribosomal internal transcribed spacer region (ITS) was amplified with the primer pairs ITS4/ITS5 (White et al. 1990). The nuclear ribosomal small and large subunit (nr*SSU* and nr*LSU*) were amplified with the primer pairs nr*SSU*-CoF/nr*SSU*-CoR and LR5/LR0R, respectively (Vilgalys and Hester 1990; Rehner and Samuels 1994; Wang et al. 2015a). The translation elongation factor 1α (*tef‐1α*) was amplified with the primers EF1α‐EF and EF1α‐ER (Bischof et al. 2006; Sung et al. 2007). The largest and second subunits of RNA polymerase II (*rpb1* and *rpb2*) were amplified with the primers RPB1‐5′F/RPB1‐5′R and RPB2-5′F/RPB2-5′R, respectively (Bischof et al. 2006; Sung et al. 2007). The polymerase chain reaction (PCR) matrix was performed in a final volume of 50 µl and the detailed information was described by Wang et al. (2022). Amplification reactions were performed in the BIORAD T100TM thermal cycler (BIO-RAD Laboratories, Hercules, CA, United States). The PCR reactions followed the procedures of Wang et al. (2015b) and the PCR products were sequenced by the Beijing Genomics Institute (Chongqing, China).

### ﻿Phylogenetic analyses

Based on the six-locus molecular, including ITS, nr*SSU*, nr*LSU*, *tef‐1α*, *rpb1* and *rpb2*, phylogenetic analyses were performed using datasets retrieved from GenBank and those generated in this work. The DNA sequences newly generated have been submitted to GenBank. The sequences downloaded from the GenBank database were based on a previous study by Hou et al. (2023) and Leão et al. (2024). The taxonomic information and corresponding GenBank accession numbers used are provided in Table 1. Sequences were aligned with MEGA v.6.06 and used to remove poorly-aligned regions and for manual adjustment (Tamura et al. 2013). Six-locus molecular were concatenated together using Phylosuite v.1.2.2 (Zhang et al. 2020). The Maximum Likelihood (ML) tree was performed using IQ-tree v.2.1.3 and the Bayesian Inference (BI) tree was performed using MrBayes v.3.2.2 (Ronquist et al. 2012; Nguyen et al. 2015). The best-fitting likelihood model for BI and ML analyses was selected using ModelFinder (Kalyaanamoorthy et al. 2017). In the phylogenetic tree of *Amphichorda* and some other genera, the TN+F+I+G4 model was selected as the optimal model for the ML analyses, with 5000 ultrafast bootstraps (Hoang et al. 2017) in a single run. The GTR+F+I+G4 model was selected as the optimal model for the BI analysis and the four Markov Chain Monte Carlo chains run for 2 million generations from a random start tree with a sampling frequency of 100 generations, in which the initial 25% of sampled data were discarded as burn-in. Phylogenetic trees were visualised in FigTree v.1.4.3 and edited in Adobe Illustrator CS6. The values of ML bootstrap proportions (BP) (≥ 70%) and the BI posterior probability (PP) (≥ 0.70) are indicated at the nodes (BP/PP).

**Table 1.** Species information and corresponding GenBank accession numbers of *Amphichorda* and close relative genera used in this study.

Species	Strain	ITS	nr*SSU*	nr*LSU*	*tef‐1α*	*rpb1*	*rpb2*
*Alloacremoniumhumicola*	CBS 613.82	NR_189433	–	NG_229089	OQ470786	–	OQ453888
*Alloacremoniumferrugineum*	CBS 102877	NR_189432	–	NG_228721	OQ470785	–	OQ453887
*Amphichordacavernicola*	CGMCC3.19571	MK329056	–	MK328961	MK335997	–	–
*Amphichordacavernicola*	LC12481	MK329057	–	MK328962	MK335998	–	–
*Amphichordacavernicola*	LC12553	MK329059	–	MK328964	MK336000	–	–
*Amphichordacavernicola*	LC12560	MK329061	–	MK328966	MK336002	–	–
*Amphichordacoprophila*	CBS 247.82 ^T^	MH861494	–	MH873238	OQ954487	–	–
*Amphichordacoprophila*	CBS 424.88	OQ942929	–	OQ943166	OQ954488	–	–
***Amphichordaexcrementa***	**YFCC AECCS848^T^**	-	**OR913433**	**OR913439**	**OR917446**	**OR917451**	**OR917443**
*Amphichordafelina*	CBS 250.34	MH855498	–	OQ943167	OQ954490	–	–
***Amphichordafelina***	**CBS 250.34**	-	**OR913436**	**OR913440**	**OR917447**	**OR917450**	**OR917444**
*Amphichordafelina*	CBS 648.66	OQ942930	–	MH870575	OQ954491	–	–
*Amphichordaguana*	CGMCC3.17908^T^	KU746665	KY883262	KU746711	KX855211	KY883202	KY883228
*Amphichordaguana*	CGMCC3.17909	KU746666	KY883263	KU746712	KX855212	KY883203	–
***Amphichordakunmingensis***	**YFCC AKYYH8414^T^**	-	**OR913435**	**OR913438**	**OR917448**	**OR917452**	–
***Amphichordakunmingensis***	**YFCC AKYYH8487**	-	**OR913434**	**OR913437**	**OR917449**	**OR917453**	**OR917445**
*Amphichordalittoralis*	FMR 17952	OQ942925	–	OQ943162	OQ954483	–	–
*Amphichordalittoralis*	FMR 19404^T^	OQ942924	–	OQ943161	OQ954482	–	–
*Amphichordalittoralis*	FMR 19611	OQ942926	–	OQ943163	OQ954484	–	–
*Amphichordamonjolensis*	COAD 3124	OQ288256	–	OQ288260	OR454090	–	OQ405040
*Amphichordamonjolensis*	COAD 3125	OQ288257	–	–	–	–	OQ405041
*Amphichordamonjolensis*	COAD 3120	OQ288258	–	–	–	–	OQ405042
*Amphichordayunnanensis*	KUMCC 21-0414	ON426823	–	–	OR025977	OR022016	OR022041
*Amphichordayunnanensis*	KUMCC 21-0415	ON426824	–	–	OR025976	OR022015	OR022040
*Amphichordayunnanensis*	KUMCC 21-0416^T^	-	–	–	OR025975	OR022014	OR022039
*Bulbitheciumammophilae*	CBS 178.78	NR_189437	–	NG_242039	OQ470793	–	OQ453895
*Bulbitheciumarxii*	CBS 737.84	NR_145040	–	HQ232159	OQ470794	–	OQ451834
*Bulbitheciumborodinense*	CBS 101148	OQ429506	–	HQ232003	–	–	–
*Bulbitheciumellipsoideum*	CBS 993.69	NR_189438	–	NG_242040	OQ470796	–	OQ453896
*Bulbitheciumhyalosporum*	CBS:318.91	MH862256	AF096172	OQ055419	OQ470797	–	OQ453897
*Bulbitheciumpinkertoniae*	CBS 157.70	NR_159611	NG_062816	NG_058554	OQ470799	–	OQ453898
*Bulbitheciumspinosum*	CBS 136.33	OQ429512	NG_062819	NG_056971	OQ470802	–	OQ453899
*Bulbitheciumtruncatum*	CBS 113718	NR_189439	–	NG_242041	OQ470803	–	OQ453900
*Clavicepspurpurea*	SA cp11	-	EF469122	EF469075	EF469058	EF469087	EF469105
*Geosmithialavendula*	CBS 344.48	MH856380	–	MH867927	–	–	–
*Geosmithiapallidum*	CBS 260.33	OQ429599	–	OQ055509	OQ470909	–	OQ453998
*Hapsidosporachrysogena*	CBS 144.62	NR_189452	NG_062810	HQ232017	OQ470953	–	OQ454043
*Hapsidosporaflava*	CBS 596.70	NR_189453	NG_062812	NG_056983	OQ470957	–	OQ454047
*Hapsidosporaglobosa*	CBS 512.70	NR_160124	–	NG_064081	OQ470963	–	OQ454053
*Hapsidosporainversa*	CBS 517.70	NR_189454	–	OQ055565	OQ470967	–	OQ454057
*Hapsidosporairregularis*	CBS 510.70	NR_160123	–	MH871595	OQ470968	–	OQ454058
*Hapsidosporastercoraria*	CBS 516.70	OQ429662	–	OQ055568	OQ470970	–	OQ454060
*Hapsidosporavariabilis*	CBS 100549	NR_189456	–	NG_229091	OQ470971	–	OQ454061
*Myriogenosporaatramentosa*	AEG 96-32	-	AY489701	AY489733	AY489628	AY489665	DQ522455
*Ovicilliumsubglobosum*	CBS 101963	NR_154335	–	NG_069329	OQ471085	–	OQ454170
*Ovicilliumattenuatum*	CBS 399.86	NR_154333	–	NG_229092	OQ471083	–	OQ454168
*Proxiovicilliumblochii*	CBS 427.93	-	HQ232182	HQ232001	–	–	–
*Proxiovicilliumlepidopterorum*	CBS 101239	NR_189482	–	NG_242070	OQ471145		OQ454214
*Proliferophialisapiculata*	CBS 303.64	NR_189480	–	NG_242064	OQ471122	–	OQ454207
*Proliferophialisapiculata*	CBS 397.78	OQ429798	–	OQ055694	–	–	OQ454209
*Stilbocreamacrostoma*	CBS 141849	OQ429874	–	OQ430123	–	–	OQ454273
*Stilbocreawalteri*	CBS 144627	NR_160063	–	NG_242075	–	–	–
*Waltergamsiaparva*	CBS 381.70A	NR_163808	–	NG_242083	OQ471279	–	OQ454346
*Waltergamsiapilosa*	CBS 124.70	NR_163809	–	OQ430199	OQ471282	–	OQ454349

Boldface: data generated in this study; ^T^: ex-type culture. A.E.G: A. E. Glenn personal collection; CBS: the culture collection of the Westerdijk Fungal Biodiversity Institute (WI); CGMCC: the China General Microbiological Culture Collection Center; COAD: the Laboratório de Micologia e Etiologia de Doenças Fúngicas de Plantas and Coleção Octávio Almeida Drummond; FMR: the culture collection of the Faculty of Medicine in Reus; KUMCC: the Kunming Culture Collection; LC: personal culture collection held in the lab of Dr Lei Cai; YFCC: the Yunnan Fungal Culture Collection (YFCC) of Yunnan University.

## ﻿Results

### ﻿Sequencing and phylogenetic analyses

**Figure 1.**
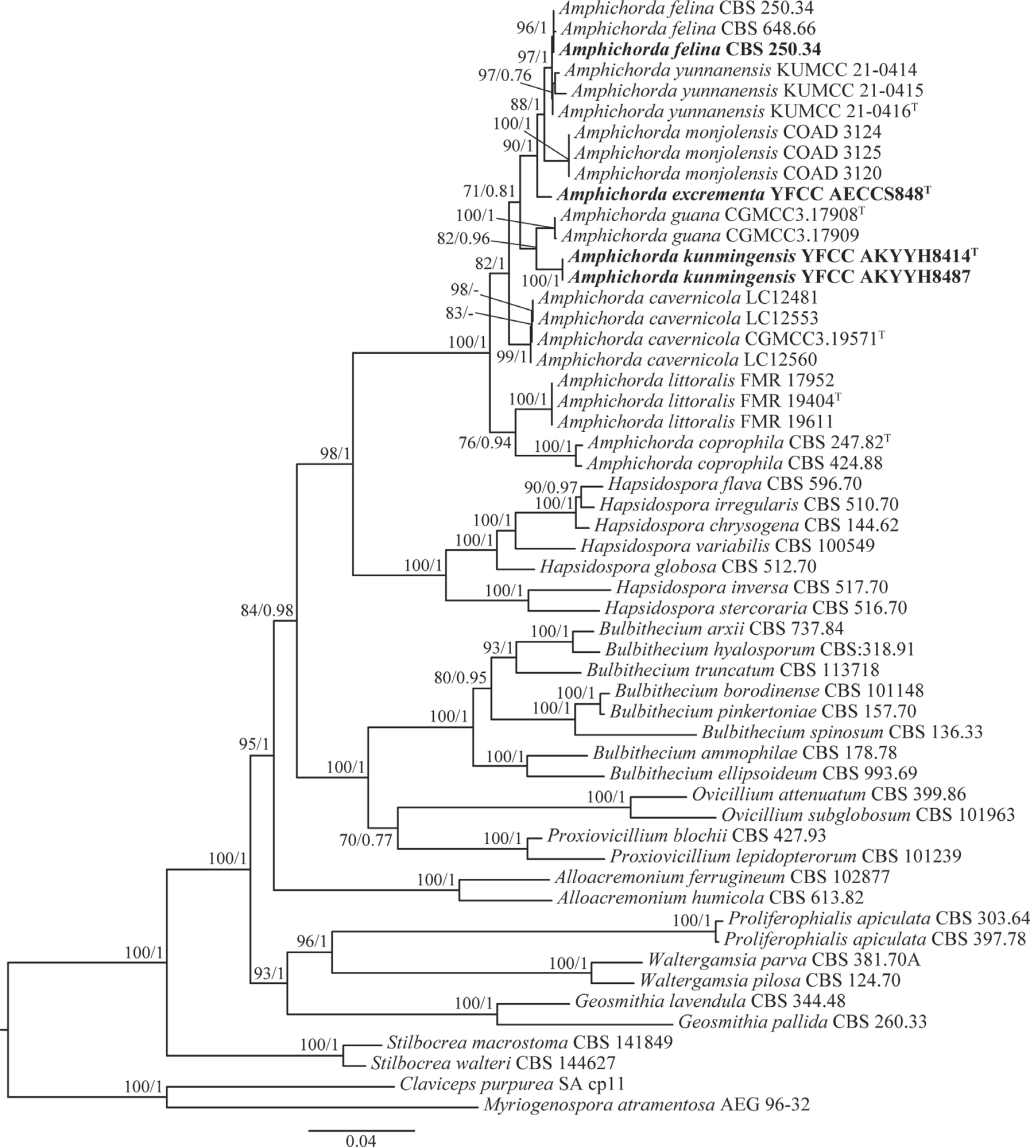
Phylogenetic tree of *Amphichorda* and close relative genera was constructed, based on Maximum Likelihood (ML) and Bayesian Inference (BI) analysis using six-locus molecular (ITS, nr*SSU*, nr*LSU*, *tef‐1α*, *rpb1*and *rpb2*) sequences. The values of ML bootstrap proportions (BP) (≥ 70%) and the BI posterior probability (PP) (≥ 0.70) are indicated at the nodes (BP/PP). The new taxa were highlighted in bold.

The phylogenetic tree was inferred using 54 strains of 12 genera from Bionectriaceae and Clavicipitaceae, including *Alloacremonium*, *Amphichorda*, *Bulbithecium*, *Claviceps*, *Geosmithia*, *Hapsidospora*, *Myriogenospora*, *Ovicillium*, *Proxiovicillium*, *Proliferophialis*, *Stilbocrea* and *Waltergamsia*. Two strains (*Clavicepspurpurea* SA cp11 and *Myriogenosporaatramentosa* AEG 96-32) of Clavicipitaceae were selected as the outgroup. The final length of the six-locus molecular sequence concatenated dataset was 5,798 bp, including 766 bp for ITS, 1,391 bp for nr*SSU*, 859 bp for nr*LSU*, 850 bp for *tef‐1α*, 781 bp for *rpb1* and 1,151 bp for *rpb2*. Phylogenetic trees from the BI and ML analyses exhibited similar topologies that had ten recognised, statistically well‐supported clades in Bionectriaceae. The four strains were clustered in the genus *Amphichorda* based on the phylogenetic analyses of the combined dataset (Fig. 1). Our ML and BI analyses showed that two new species (i.e. *A.excrementa* and *A.kunmingensis*) and one known species were recognised. The new species, *A.excrementa* and *A.kunmingensis*, were well-supported by bootstrap proportions (BP = 90% and BP = 82%, respectively) and posterior probabilities (PP = 1.00 and PP = 0.96, respectively).

### ﻿Taxonomy

#### ﻿ Amphichorda excrementa

Taxon classificationFungiHypocrealesBionectriaceae

Hong Yu bis, Z.Q. Wang, Q.Y. Dong & Y. Wang sp. nov.

DBF78251-A66F-5FD7-AD9D-8DF84A7E84EE

 851377

Fig. 2

##### Etymology.

Refers to the excrement material from which this fungus was isolated.

**Figure 2.**
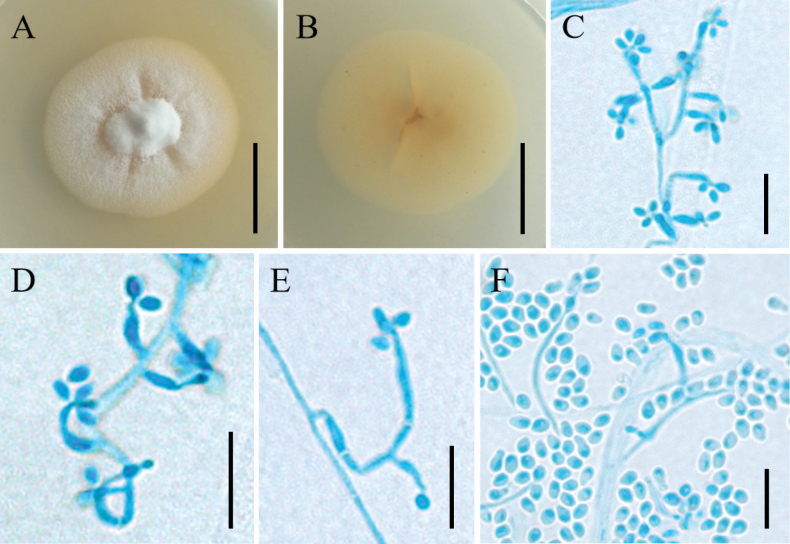
Morphology of *Amphichordaexcrementa***A, B** colony character on PDA medium after 30 d (**A** obverse **B** reverse) **C–F** conidiophores, conidiogenous cells and conidia. Scale bars: 2 cm (**A, B**); 10 µm (**C–F**).

##### Type.

China, Yunnan Province, Kunming City, Changchongshan Country Park, 11 July 2019, Hong Yu and Yao Wang (YHH AECCS200777, ***holotype***; YFCC AECCS848, ex-type).

##### Description.

***Sexual morph***: Undetermined. ***Asexual morph***: Colonies on PDA attaining a diameter of 42–44 mm after a month at 25 °C, white to cream, with high mycelial density, cottony, with a yellow margin, reverse pale yellow. ***Hyphae*** branched, smooth-walled, septate, hyaline, 0.6–1.3 µm wide. Cultures readily produced phialides and conidia after 3 weeks on potato dextrose agar at room temperature. ***Conidiophores*** arising laterally from hyphae, cylindrical, straight or slightly curved, hyaline and occasionally branched. ***Phialides*** arising laterally from aerial hyphae, occasionally solitary, mostly in whorls of 2–3 on lateral branches from the mycelia, basal portion cylindrical or flask-shaped, usually curved, 4.1–13.9 × 1.3–2.1 µm, tapering abruptly towards the apex, have a distinctly thin neck. ***Conidia*** 1.7–3.0 × 1.2–2.5 µm, one-celled, smooth-walled, hyaline, globose to elliptical, single. ***Chlamydospores*** not observed.

##### Substrate.

Animal faeces.

##### Distribution.

China.

##### Commentary.

Phylogenetic analyses showed that *Amphichordaexcrementa* formed a separate clade with statistical support from the BI posterior probabilities (PP = 1.00) and the ML bootstrap proportions (BP = 90%) and was closely related to *A.felina*, *A.yunnanensis* and *A.monjolensis*. However, *A.excrementa* can be distinguished from three species by morphological differences. The phialides of *A.excrementa* were longer (4.1–13.9 × 1.3–2.1 µm) than those of *A.felina* (1.5–8.5 × 1.8–2.9 µm) and the conidia were smaller than those of *A.felina* (1.7–3.0 × 1.2–2.5 µm vs. 2.5–4.7 × 2–3.5 µm). The phialides of *A.excrementa* were longer (4.1–13.9 × 1.3–2.1 µm) than those of *A.yunnanensis* (4–12 × 1–4 µm) and the conidia were smaller than those of *A.felina* (1.7–3.0 × 1.2–2.5 µm vs. 2–5 × 2–4 µm). The conidia of *A.monjolensis* were longer than those of *A.excrementa* (2.8–3.7 × 1.8–2.9 µm vs. 1.7–3.0 × 1.2–2.5 µm).

#### ﻿ Amphichorda felina

Taxon classificationFungiHypocrealesBionectriaceae

(DC.) Fr., Syst. orb. veg. (Lundae) 1: 170 (1825).

D064A027-4CEB-5360-85A4-0286C61AC039

 562082

Fig. 3

##### Description.

The morphological description of this study is based on the specimen, CBS 250.34. ***Sexual morph***: Undetermined. ***Asexual morph***: Colonies on PDA attaining a diameter of 36–38 mm after a month at 25 °C, white to creamy-white, hard texture, felt-like, reverse black-brown, many conidia assemble to form powder. ***Hyphae*** branched, smooth-walled, septate, hyaline, 1.2–2.4 µm wide. ***Phialides*** arising laterally from aerial hyphae, erect or irregularly curved, 1.5–4.1 × 1.8–2.9 µm. ***Conidia*** 2.9–4.7 × 2.4–3.5 µm, one-celled, smooth-walled, hyaline, broadly ellipsoid or subglobose, single or aggregated into spheres. ***Chlamydospores*** not observed.

**Figure 3.**
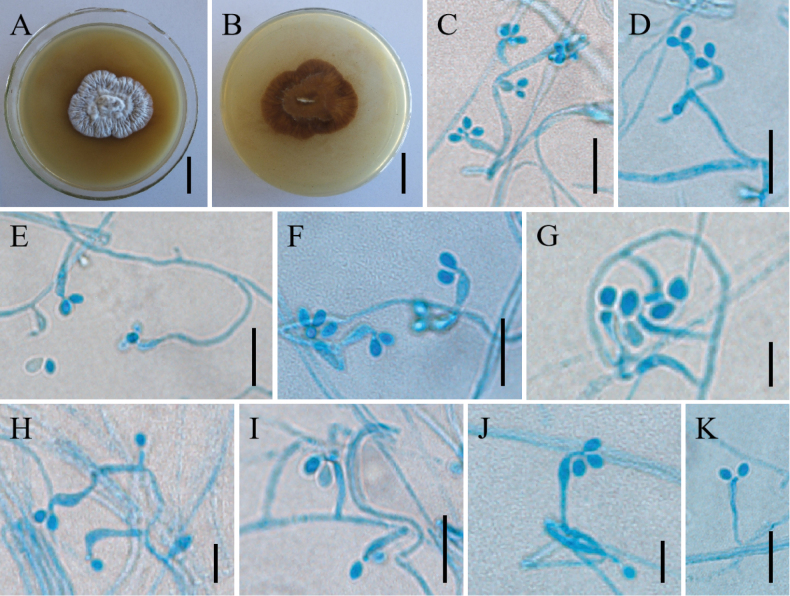
Morphology of *Amphichordafelina***A, B** colony character on PDA medium after 30 d (**A** obverse **B** reverse) **C–K** conidiophores, conidiogenous cells and conidia. Scale bars: 2 cm (**A, B**); 10 µm (**C–F, I, K**); 5 µm (**G–H, J**).

##### Substrate.

Pupa of *Anaitisefformata*, rabbit dung, mouldy leaves, porcupine dung, cat dung.

##### Distribution.

Argentina, Britain, France, Germany.

##### Commentary.

Guerra-Mateo et al. (2023) proposed that the strain CBS 250.34 can be accepted as a reference to stabilise the nomenclature of *Amphichordafelina* and thus the genus *Amphichorda*, but should be avoided to indicate it as a type strain. In this study, the strain, CBS 250.34, was available in the CBS culture collection and morphological observations were made. Its morphology was generally consistent with those described by De Hoog (1972), with one difference being that this study extended the phialides (1.5–8.5 × 1.8–2.9 µm) and conidia size range of this species (2.5–4.7 × 2–3.5 µm).

#### ﻿ Amphichorda kunmingensis

Taxon classificationFungiHypocrealesBionectriaceae

Hong Yu bis, Z.Q. Wang, Q.Y. Dong & Y. Wang sp. nov.

8BB759EA-C045-52A6-9308-3D269DFD9A6B

 851378

Fig. 4

##### Etymology.

Named from the location Kunming City where the species was collected.

##### Type.

China, Yunnan Province, Kunming City, Wild Duck Lake Forest Park, 16 July 2019, Hong Yu and Yao Wang (YHH AKYYH200704, ***holotype***; YFCC AKYYH8414, ex-type).

**Figure 4.**
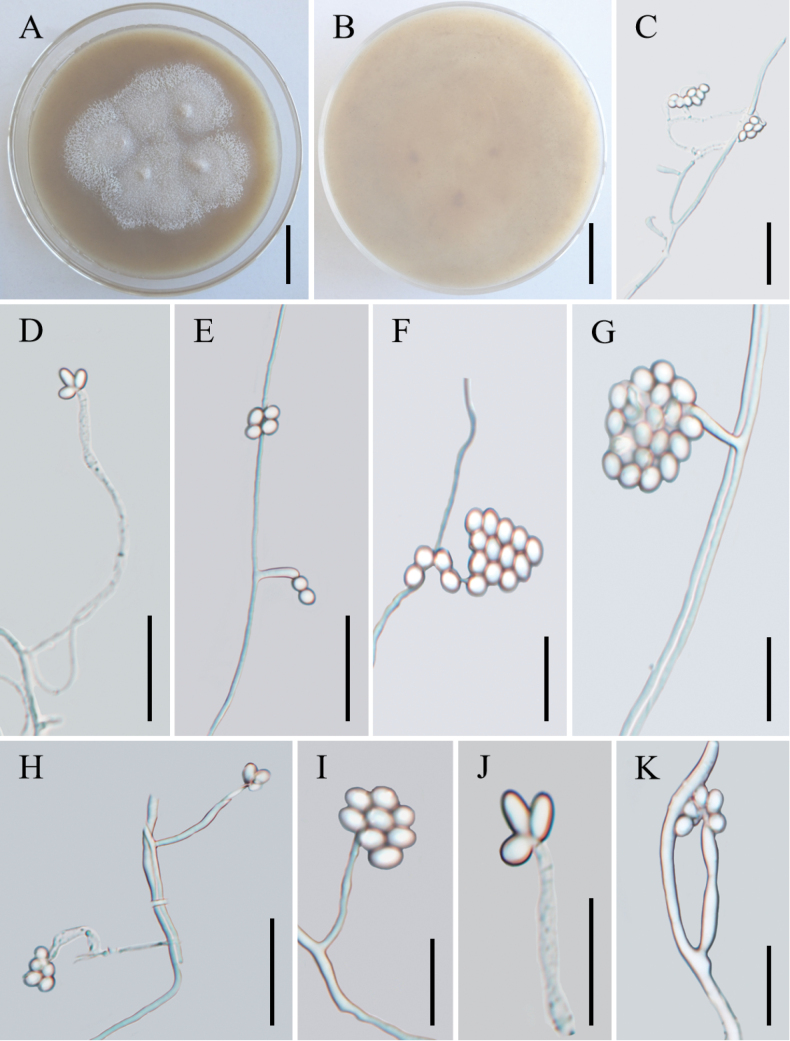
Morphology of *Amphichordakunmingensis***A, B** colony character on PDA medium after 30 d (**A** obverse **B** reverse) **C–K** conidiogenous cells and conidia. Scale bars: 2 cm (**A, B**); 20 µm (**C–E, H**); 10 µm (**F–G, I–K**).

##### Description.

***Sexual morph***: Undetermined. ***Asexual morph***: Colonies on PDA attaining a diameter of 52–54 mm after a month at 25 °C, white to pale grey, with low mycelial density, lanose. ***Hyphae*** hyaline, branched, smooth-walled, septate, 0.7–1.9 µm wide. Cultures readily produced phialides and conidia after 3 weeks on potato dextrose agar at room temperature. ***Phialides*** arising laterally from aerial hyphae, solitary, occasionally in simple whorls on lateral branches from the mycelia, basal portion cylindrical or fusiform, straight or irregularly bent, 6.1–17.5 × 1.4–2.9 µm. ***Conidia*** 2.3–4.2 × 1.6–3.0 µm, one-celled, smooth-walled, hyaline, globose to elliptical, single or aggregating in small heads at the apex of conidiogenous cells. ***Chlamydospores*** not observed.

##### Substrate.

Animal faeces.

##### Distribution.

China.

##### Other material examined.

China, Yunnan Province, Kunming City, Wild Duck Lake Forest Park, 16 July 2019, Hong Yu and Yao Wang (YHH AKYYH200776, paratype; YFCC AKYYH8487, ex-paratype).

##### Commentary.

Three species of *Amphichorda* were from China and *A.yunnanensis* was distributed in Yuxi City, Yunnan Province. The two new species in this study were from Kunming City, Yunnan Province. According to the phylogenetic tree, the new species, *A.kunmingensis*, forms a separate branch in *Amphichorda* and is sister to *A.guana*. However, it differs from *A.guana* by its smaller conidia. Although *A.kunmingensis*, *A.excrementa* and *A.yunnanensis* were all collected from Yunnan, their morphology was quite different (see Table 2). *Amphichordakunmingensis* differs from *A.excrementa* in its usually curved and longer phialides (6.1–17.5 × 1.4–2.9 μm vs. 4.1–13.9 × 1.3–2.1 μm) and larger conidia (2.3–4.2 × 1.6–3.0 μm vs. 1.7–3.0 × 1.2–2.5 μm). *Amphichordakunmingensis* differs from *A.yunnanensis* in the shape of its phialides and narrower conidia.

**Table 2.** Geographical location, hosts/substrates and asexual morphology of *Amphichorda*.

Species	Country	Host/Substrate	Conidiophores	Phialides (μm)	Conidia (μm)	References
*Amphichordacavernicola*	China	Bird faeces; soil; plant debris; animal faeces; bat guano	Cylindrical, straight or slightly curved, occasionally branched	Fusiform or ellipsoidal, straight or irregularly bent, 4.5–8.0 × 2.0–3.0	Broadly ellipsoidal to subglobose, 2.5–4.0 × 2.0–3.5	Zhang et al. (2021)
*A.coprophila*	Canada; England	Chipmunk, rabbit and porcupine dung	Straight or flexuous, unbranched, bearing lateral or terminal conidiogenous cells, arranged singly or in whorls	Flask-shaped, usually with a strongly bent neck, 6–10 × 2–2.5	Subglobose to somewhat ellipsoidal, 3.5–5.5 × 2–3	Guerra-Mateo et al. (2023)
***A.excrementa***	**China**	Animal faeces	**Cylindrical, straight or slightly curved, occasionally branched**	**Occasionally solitary, mostly in whorls of 2–3, basal portion cylindrical or flask-shaped, usually curved, 4.1–13.9 × 1.3–2.1**	**Globose to elliptical 1.7–3.0 × 1.2–2.5**	**In this study**
*A.felina*	Britain, Germany, Argentina, France	Pupa of *Anaitisefformata*; rabbit dung; mouldy leaves; porcupine dung; cat dung	Straight	Solitarily or in small groups, consisting of a swollen, flask-shaped or curved, occasionally elongate basal part, 1.5–8.5 × 1.8–2.9	Subglobose, ellipsoidal or ovoidal, sometimes with a pointed base, 2.5–4.7 × 2–3.5	De Hoog (1972); **In this study**
*A.guana*	China	Bat guano	Straight or slightly curved	Fusiform or ellipsoidal, straight or irregularly bent, 7–10 × 2–3	Broadly ellipsoid to subglobose, 4.5–5.5 × 3.5–5	Zhang et al. (2017)
***A.kunmingensis***	**China**	**Animal faeces**	-	**Solitary, occasionally in simple whorls, basal portion cylindrical or fusiform, straight or irregularly bent, 6.1–17.5 × 1.4–2.9**	**Globose to elliptical 2.3–4.2 × 1.6–3.0**	**In this study**
*A.littoralis*	Spain	Sediments; fragment of floating rubber tire	Straight or flexuous, commonly unbranched, bearing lateral or terminal conidiogenous cells, arranged singly or in whorls of 2–4	Flask-shaped, usually with a strongly bent neck, 6–10 (–11.5) × 1.5–2	Subglobose, 3–4 × 2.5–3	Guerra-Mateo et al. (2023)
*A.monjolensis*	Brazil	on PDA plate consumed by an insect	Cylindrical, bearing one or more conidiogenous cells, straight or slightly bent, solitary or synnematous, sometimes branched	Flask-shaped, straight or irregularly bent, 3.1–6.1 × 2.7–5.1	Holoblastic, 2.8–3.7×1.8–2.9	Leão et al. (2024)
*A.yunnanensis*	China	Wing surfaces of *Rhinolophus*	Cylindrical, straight or slightly curved, branched	Monoblastic to polyblastic, ampulliform to flask-shaped, 4–12 × 1–4	Globose to oval, slightly ellipsoid, 2–5 × 2–4	Liu et al. (2023)

## ﻿Discussion

The phylogenetic analyses, based on the five-gene (nr*SSU*, nr*LSU*, *tef‐1α*, *rpb1* and *rpb2*) sequence and ITS data were conducted and *Amphichordaexcrementa* and *A.kunmingensis* were introduced. The morphological characteristics of the new species are similar to those of other *Amphichorda* species. Its conidiophores straight or slightly curved; phialides solitary, simple whorls or several whorls, straight or irregularly bent, usually curved, tapering abruptly towards the apex; conidia solitary or clumped, one-celled, shape variable (Table 2). They were similar to those of *Beauveria* and all species of *Amphichorda* do not have the elongate conidiogenous cells with apical denticulate rachis that are characteristic of *Beauveria*.

The species of *Amphichorda* has an extremely wide distribution, including Argentina, Canada, China, France, Germany, Great Britain, Spain (Table 2). Amongst the *Amphichorda* species, *A.felina*, *A.cavernicola*, *A.guana* and *A.monjolensis* were found in caves, especially *A.felina*, which was widely distributed in caves (Vanderwolf et al. 2013; Zhang et al. 2017, 2021; Vanderwolf et al. 2018; Leão et al. 2024). *Amphichordalittoralis* was found in Mediterranean coast sediments at 20 m depth (Guerra-Mateo et al. 2023). In contrast to the particular ecology of caves and the sea, *A.coprophila* was isolated from rabbit, chipmunk and porcupine dung and *A.yunnanensis* was isolated from the wing surfaces of *Rhinolophusaffinis* (Guerra-Mateo et al. 2023; Liu et al. 2023). *Amphichordaexcrementa* and *A.kunmingensis* were isolated from animal faeces in the Park. The substrates of *Amphichorda* were complex and varied, being mainly animal faeces, i.e. bird, cat, bat, chipmunk, rabbit and porcupine dung, but they have also been isolated in the pupa of *Anaitisefformata*, mouldy leaves, plant debris, sediments, fragments of floating rubber tyres, wing surfaces of *Rhinolophus* and soil. Most species of the genus *Amphichorda* have been isolated on animal faeces and are quite unique to their parasitic environments. This is unique to the biological characteristics and ecological habits for the genus *Amphichorda*.

Coprophilous fungi, particularly coprophilous ascomycetes, will be a rich source of antibiotics and other biologically important secondary metabolites (Bills et al. 2013). Species of the genus *Amphichorda* tend to have special physiological and metabolic characteristics due to the uniqueness of their growth environment. Additionally, some of their species have been reported to have high application value, such as *A.felina*, which was a well-known producer of insecticidal cyclodepsipeptide and cyclosporin C (Langenfeld et al. 2011; Chung et al. 2013; Xu et al. 2018). Furthermore, the study by Liang et al. (2021) successfully established a genetic transformation system in *A.guana* strain LC5815, which facilitated the development of bioactive secondary metabolites in fungi. Two new species of the genus *Amphichorda*, described in the present study, were isolated from animal faeces and may have good potential for natural product research.

## Supplementary Material

XML Treatment for Amphichorda excrementa

XML Treatment for Amphichorda felina

XML Treatment for Amphichorda kunmingensis

## ﻿Additional information

### Author ORCIDs

Zhi-Qin Wang https://orcid.org/0000-0001-9022-3635

Hong Yu https://orcid.org/0000-0002-2149-5714

### Data availability

All of the data that support the findings of this study are available in the main text.

## References

[B1] Araújo JPM Lebert BM Vermeulen S Brachmann A Ohm RA Evans HC de Bekker C (2022) Masters of the manipulator: Two new hypocrealean genera, *Niveomyces* (Cordycipitaceae) and *Torrubiellomyces* (Ophiocordycipitaceae), parasitic on the zombie ant fungus *Ophiocordycepscamponoti*-*floridani*. Persoonia 49(1): 171–194. 10.3767/persoonia.2022.49.05 38234384 PMC10792228

[B2] Belyagoubi L Belyagoubi-Benhammou N Jurado V Dupont J Lacoste S Djebbah F Ounadjela FZ Benaissa S Habi S Abdelouahid DE Saiz-Jimenez C (2018) Antimicrobial activities of culturable microorganisms (Actinomycetes and Fungi) isolated from Chaabe Cave, Algeria. International Journal of Speleology 47(2): 189–199. 10.5038/1827-806X.47.2.2148

[B3] Bills GF Gloer JB An Z (2013) Coprophilous fungi: Antibiotic discovery and functions in an underexplored arena of microbial defensive mutualism. Current Opinion in Microbiology 16(5): 549–565. 10.1016/j.mib.2013.08.001 23978412

[B4] Bischof JF Rehner SA Humber RA (2006) *Metarhiziumfrigidum* sp. nov.: A cryptic species of *M.anisopliae* and a member of the *M.favoviride* Complex. Mycologia 98(5): 737–745. 10.1080/15572536.2006.11832645 17256577

[B5] Carmichael JW Kendrick WB Conners IL Sigler L (Eds) (1980) Genera of Hyphomycetes. University of Alberta Press.

[B6] Chung YM El-Shazly M Chuang DW Hwang TL Asai T Oshima Y Ashour ML Wu YC Chang FR (2013) Suberoylanilide hydroxamic acid, a histone deacetylase inhibitor, induces the production of anti-inflammatory cyclodepsipeptides from *Beauveriafelina*. Journal of Natural Products 76(7): 1260–1266. 10.1021/np400143j 23822585

[B7] De Hoog GS (1972) The Genera *Beauveria*, *Isaria*, *Tritirachium* and *Acrodontium* gen. nov. Studies in Mycology 1: 1–41.

[B8] Fries EM (1825) Systema orbis vegetabilis: Primas Lineas Novae Constructionis Periclitatur; E Typographia Academica. Lunde, Norway 1: 170.

[B9] Guerra-Mateo D Gené J Baulin V Cano-Lira JF (2023) Phylogeny and Taxonomy of the Genus *Amphichorda* (Bionectriaceae): An Update on *Beauveria*-like Strains and Description of a Novel Species from Marine Sediments. Diversity 15(7): 795. 10.3390/d15070795

[B10] Hoang DT Chernomor O von Haeseler A Minh BQ Vinh LS (2017) UFBoot2: Improving the ultrafast bootstrap approximation. Molecular Biology and Evolution 35(2): 518–522. 10.1093/molbev/msx281 PMC5850222 29077904

[B11] Hou LW Giraldo A Groenewald JZ Rämä T Summerbell RC Huang GZ Cai L Crous PW (2023) Redisposition of acremonium-like fungiin Hypocreales. Studies in Mycology 105(1): 23–203. 10.3114/sim.2023.105.02 38895703 PMC11182610

[B12] Kalyaanamoorthy S Minh BQ Wong TKF von Haeseler A Jermiin LS (2017) ModelFinder: Fast model selection for accurate phylogenetic estimates. Nature Methods 14(6): 587–589. 10.1038/nmeth.4285 28481363 PMC5453245

[B13] Lamarck JB (1815) Flore Françoise ou Descriptions Succinctes de Toutes les Plantes Qui Croissent Naturellement en France, Disposées Selon Une Nouvelle Méthode D’Analyse, et Précédées Par un Exposé des Principes Élémentaires de la Botanique. Libraire Chez Desray, Paris, vol. 6, 30 pp. 10.5962/bhl.title.112968

[B14] Langenfeld A Blond A Gueye S Herson P Nay B Dupont J Prado S (2011) Insecticidal cyclodepsipeptides from *Beauveriafelina*. Journal of Natural Products 74(4): 825–830. 10.1021/np100890n 21438588

[B15] Leão AF Condé TO Dutra YLG Rosado AWC Grazziotti PH Neves SC Fraga LMS Kasuya MCM Pereira OL (2024) *Amphichordamonjolensis* sp. nov., a new fungal species isolated from a Brazilian limestone cave, with an update on acremonium-like species in Bionectriaceae. Brazilian Journal of Microbiology. 10.1007/s42770-024-01289-y PMC11153450 38462595

[B16] Lendemer JC (2020) Epitypes are forever: Best Practices for an Increasingly Misused Nomenclatural Action. Taxon 69(5): 849–850. 10.1002/tax.12289

[B17] Liang M Li W Qi D Chen C Cai L Yin B. (2021) Establishment of a genetic transformation system in guanophilic fungus *Amphichordaguana*. Journal of Fungi 7: 138. 10.3390/jof7020138 PMC7918455 33672933

[B18] Liu M Hodge KT (2005) *Hypocrellazhongdongii* sp. nov., the teleomorph of *Aschersoniaincrassata*. Mycological Research 109: 818–824. 10.1016/10.1017/S095375620500290X 16121568

[B19] Liu XF Tibpromma S Hughes AC Chethana KWT Wijayawardene NN Dai DQ Du TY Elgorban AM Stephenson SL Suwannarach N Xu JC Lu L Xu RF Maharachchikumbura SSN Zhao CL Bhat DJ Sun YM Karunarathna SC Mortimer PE (2023) Culturable mycota on bats in central and southern Yunnan Province, China. Mycosphere 14(1): 497–662. 10.5943/mycosphere/14/1/7

[B20] Nguyen LT Schmidt HA von Haeseler A Minh BQ (2015) IQ-TREE: A fast and efective stochastic algorithm for estimating maximum likelihood phylogenies. Molecular Biology and Evolution 32(1): 268–274. 10.1093/molbev/msu300 25371430 PMC4271533

[B21] Rehner SA Samuels GJ (1994) Taxonomy and phylogeny of *Gliocladium* analysed from nuclear large subunit ribosomal DNA sequences. Mycological Research 98(6): 625–634. 10.1016/S0953-7562(09)80409-7

[B22] Rehner SA Minnis AM Sung GH Luangsa-ard JJ Devotto L Humber RA (2011) Phylogeny and systematics of the anamorphic, entomopathogenic genus *Beauveria*. Mycologia 103(5): 1055–1073. 10.3852/10-302 21482632

[B23] Ronquist F Teslenko M van der Mark P Ayres DL Darling A Höhna S Larget B Liu L Suchard MA Huelsenbeck JP (2012) MrBayes 3.2: Efcient Bayesian phylogenetic inference and model choice across a large model space. Systematic Biology 61(3): 539–542. 10.1093/sysbio/sys029 22357727 PMC3329765

[B24] Sung GH Hywel-Jones NL Sung JM Luangsa-Ard JJ Shrestha B Spatafora JW (2007) Phylogenetic classifcation of *Cordyceps* and the clavicipitaceous fungi. Studies in Mycology 57: 5–59. 10.3114/sim.2007.57.01 18490993 PMC2104736

[B25] Tamura K Stecher G Peterson D Filipski A Kumar S (2013) MEGA6: Molecular Evolutionary Genetics Analysis Version 6.0. Molecular Biology and Evolution 30(12): 2725–2729. 10.1093/molbev/mst197 24132122 PMC3840312

[B26] Vanderwolf KJ Malloch D Mcalpine DF Forbes GJ (2013) A world review of fungi, yeasts, and slime molds in caves. International Journal of Speleology 42(1): 77–96. 10.5038/1827-806X.42.1.9

[B27] Vilgalys R Hester M (1990) Rapid genetic identifcation and mapping of enzymatically amplifed ribosomal DNA from several *Cryptococcus* species. Journal of Bacteriology 172(8): 4238–4246. 10.1128/jb.172.8.4238-4246.1990 2376561 PMC213247

[B28] Wang YB Yu H Dai YD Wu CK Zeng WB Yuan F Liang ZQ (2015a) *Polycephalomycesagaricus*, a new hyperparasite of *Ophiocordyceps* sp. infecting melolonthid larvae in southwestern China. Mycological Progress 14(9): 70. 10.1007/s11557-015-1090-7

[B29] Wang YB Yu H Dai YD Chen ZH Zeng WB Yuan F Liang ZQ (2015b) *Polycephalomycesyunnanensis* (Hypocreales), a new species of *Polycephalomyces* parasitizing *Ophiocordycepsnutans* and stink bugs (Hemipteran adults). Phytotaxa 208: 34–44. 10.11646/phytotaxa.208.1.3

[B30] Wang YB Wang Y Fan Q Duan DE Zhang GD Dai RQ Dai YD Zeng WB Chen ZH Li DD Tang DX Xu ZH Sun T Nguyen TT Tran NL Dao VM Zhang CM Huang LD Liu YJ Zhang XM Yang DR Sanjuan T Liu XZ Yang ZL Yu H (2020) Multigene phylogeny of the family Cordycipitaceae (Hypocreales): New taxa and the new systematic position of the Chinese cordycipitoid fungus *Paecilomyceshepiali*. Fungal Diversity 103(1): 1–46. 10.1007/s13225-020-00457-3

[B31] Wang ZQ Wang Y Dong QY Fan Q Dao VM Yu H (2022) Morphological and Phylogenetic Characterization Reveals Five New Species of *Samsoniella* (Cordycipitaceae, Hypocreales). Journal of Fungi (Basel, Switzerland) 8(7): 747. 10.3390/jof8070747 35887502 PMC9321185

[B32] White TJ Bruns TD Lee SB Taylor JW (1990) Amplifcation and direct sequencing of fungal ribosomal RNA genes for phylogenetics. In: Innis MA Gelfand DH Sninsky JJ White TJ (Eds) PCR protocols: a guide to methods and applications.Academic, New York, 315–322. 10.1016/B978-0-12-372180-8.50042-1

[B33] Xu L Li Y Biggins JB Bowman BR Verdine GL Gloer JB Alspaugh JA Bills GF (2018) Identifcation of cyclosporin C from *Amphichordafelina* using a *Cryptococcusneoformans* differential temperature sensitivity assay. Applied Microbiology and Biotechnology 102(5): 2337–2350. 10.1007/s00253-018-8792-0 29396588 PMC5942556

[B34] Zhang ZF Liu F Zhou X Liu XZ Liu SJ Cai L (2017) Culturable mycobiota from Karst caves in China, with descriptions of 20 new species. Persoonia 39(1): 1–31. 10.3767/persoonia.2017.39.01 29503468 PMC5832949

[B35] Zhang D Gao FL Jakovlić I Zou H Zhang J Li WX Wang GT (2020) PhyloSuite: An integrated and scalable desktop platform for streamlined molecular sequence data management and evolutionary phylogenetics studies. Molecular Ecology Resources 20(1): 348–355. 10.1111/1755-0998.13096 31599058

[B36] Zhang ZF Zhou SY Eurwilaichitr L Ingsriswang S Raza M Chen Q Zhao P Liu F Cai L (2021) Culturable mycobiota from Karst caves in China II, with descriptions of 33 new species. Fungal Diversity 106(1): 29–136. 10.1007/s13225-020-00453-7

